# Supplementary material for: Yiai Fuzheng decoction inhibits triple-negative breast cancer by remodeling the immune microenvironment
Source: Front Immunol. 2025 Sep 30;16:1615631. doi: 10.3389/fimmu.2025.1615631 (PMC12518410; doi:10.3389/fimmu.2025.1615631)
Supplement: Supplementary file 2 [file DataSheet1.zip › Supplementary File 2/Treatment/C_vs_H/02_PCA/All_Groups/All_Combinaiton/PC23_Score_2D_Label.pdf]

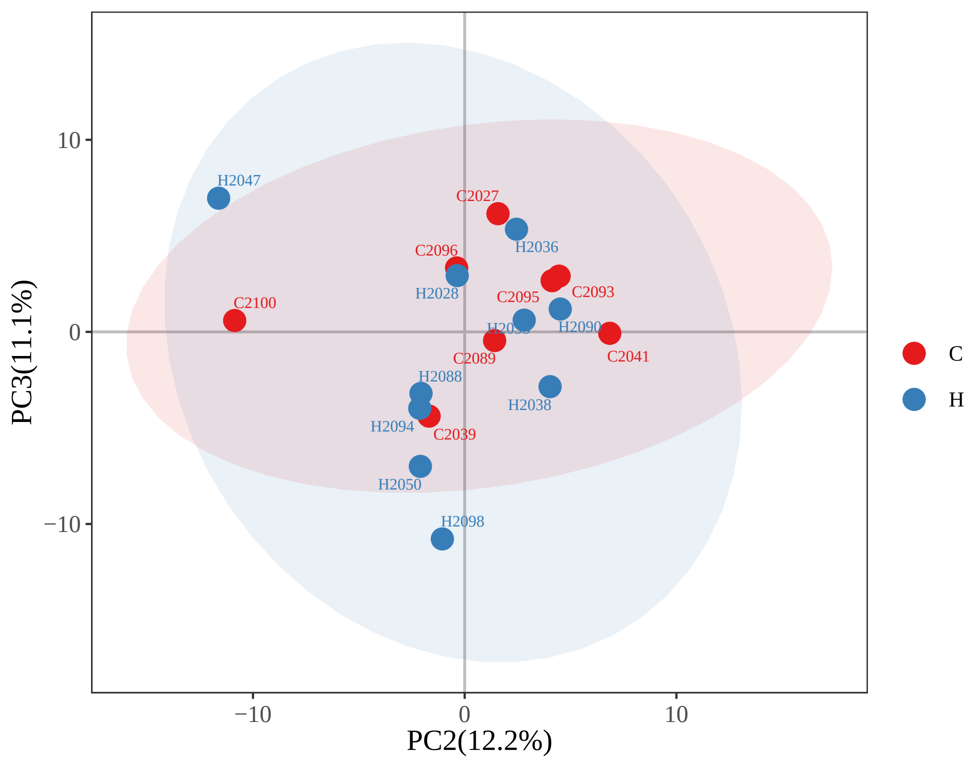
H2047
C2027
H2036
C2096
H2028
C2093
C2095
C2100
H2090
C2041
C2089
H2088
H2038
H2094
C2039
H2050
H2098
10
0
−10
−10
0
10
PC2(12.2%)
PC3(11.1%)
C
H
